# Rasch Analysis of General Self-Efficacy Among Individuals with Intellectual Disabilities

**DOI:** 10.3390/bs15121639

**Published:** 2025-11-28

**Authors:** Eun-Young Park

**Affiliations:** Department of Secondary Special Education, Jeonju University, Jeonju 55069, Republic of Korea; eunyoung@jj.ac.kr

**Keywords:** general self-efficacy scale, Rasch analysis, psychometric properties, intellectual disabilities, item difficulty

## Abstract

The self-efficacy of individuals with intellectual disabilities is considered an important factor in the psychological adjustment process. The General Self-Efficacy Scale (GSES) is commonly used to measure self-efficacy. However, previous studies have not examined the psychometric properties of the GSES among individuals with intellectual disabilities. Therefore, this study investigated the psychometric properties of the GSES using the Rasch model based on the item response theory. This study used secondary data from the Employment Panel Survey of Persons with Disabilities provided by the Republic of Korea Employment Agency for Persons with Disabilities. The panel survey collected data from individuals with intellectual disabilities in the Republic of Korea using the GSES. This study analyzed data from 232 individuals to determine GSES item fitness, item difficulty, rating scale fit, and reliability. The results revealed that eight of ten GSES items exhibited appropriate fit. Item difficulty required modification, indicating the need for items with lower difficulty. The four-point Likert scale used for responses was appropriate. Person and item separation indices demonstrated good scale reliability. These findings suggest that the GSES is effective for measuring self-efficacy in people with intellectual disabilities; however, some adjustments, such as changes in difficulty level, are required.

## 1. Introduction

The American Association on Intellectual and Developmental Disabilities defines intellectual disability (ID) as a condition characterized by significant difficulties in intellectual functioning and adaptive behavior, affecting everyday social and practical skills, with onset before age 22 (Schalock et al., 2021). Individuals with ID exhibit difficulties in intellectual functioning, including reasoning, problem solving, planning, abstract thinking, judgment, academic learning, and learning from experience (APA, 2013). Their significant deficit in adaptive skills limits their ability to carry out age-appropriate daily activities (Boat et al., 2015). Due to their inadequately developed metacognitive skills, individuals with ID have inhibited ability to memorize information, rehearse, organize materials, and systematically regulate their learning process (Erez & Peled, 2001; Hunt & Marshall, 2002).

Such cognitive and adaptive limitations often lead to repeated experiences of task failure, resulting in frustration and avoidance of new challenges, which negatively influence the development of self-efficacy. Individuals with ID are sometimes perceived as lacking motivation or overly passive in task performance (Cuskelly & Gilmore, 2014). This is attributable to anxiety and frustration stemming from repeated failures, which can hinder goal pursuit and reinforce “learned helplessness” (Gacek et al., 2017). As a result, their behaviors may be driven only by external rewards or reinforcement, rather than internal satisfaction, and they may be less likely to initiate actions or actively set goals (Bernie-Smith et al., 2002; Taylor et al., 2005). Considering these characteristics, it is important to improve the self-efficacy of individuals with ID. Self-efficacy refers to the belief in one’s ability to achieve desired goals (Bandura, 2006), exercise control over performance, and organize behavior in a specific situation (Patrick et al., 1997). Self-efficacy affects the way of thinking that determines the choice of behavior, the extent of effort exerted, the length of effort until the behavior is completed, and emotional responses (Bandura, 1977; Bandura & Schunk, 1981; Bandura et al., 1980).

Self-efficacy determines the amount of effort invested in a behavior and the degree to which the individual continues a behavior despite obstacles or aversive experiences. Therefore, stronger self-efficacy is associated with greater effort and longer duration of the selected behavior, thereby limiting other behaviors and leading to effort investment even in difficult tasks (Bandura et al., 1975). Studies have reported that high self-efficacy aids the recovery from, and treatment of, various disorders, such as phobias, obesity, smoking, and heart disease (Bandura, 1986; Litt, 1988; O’Leary, 1985). In addition to behavioral patterns, self-efficacy affects emotions. According to Bandura (1982), fear and anxiety are caused by a sense of helplessness. If the situation is controlled, fear and anxiety decrease. Self-efficacy is the belief that one can perform coping behaviors in a given situation; anxiety is negatively correlated with self-efficacy.

To date, there has been little research on self-efficacy in individuals with ID. However, studies have explored the relationship between self-efficacy and other psychological factors, such as physical activity participation. Peterson et al. (2008) used self-report scales to measure self-efficacy and social support for physical leisure activities in 152 adults with ID. They reported that social support and self-efficacy positively predicted physical activity participation, with self-efficacy mediating between social support and physical activity. Jo et al. (2018) found increases in muscular endurance, self-efficacy, and physical activity levels in 23 adults with ID following a 12-week exercise program. Relatedly, low self-efficacy levels in individuals with ID have been reported to result in low participation in programs that can benefit their physical and mental health (Stevens et al., 2018; Yu et al., 2022). Self-efficacy is an important predictor of mental health. Therefore, improving self-efficacy in individuals with ID could help to alleviate their tendency to struggle with mental health issues. Moreover, self-efficacy is closely associated with the quality of life, career paths, health, and social participation of individuals with ID (Nota et al., 2010; Peterson et al., 2008).

To accurately understand and predict the level of self-efficacy, the measurement scale must be reliable and valid, and knowledge and understanding of the scale characteristics must be supported by empirical research. The Glasgow Social Self-Efficacy Scale (GSSES), developed to assess social self-efficacy in individuals with ID, has shown strong test–retest reliability, acceptable internal reliability, and low concurrent validity (Payne & Jahoda, 2004). Thus, although larger studies are required to examine its psychometric properties, the GSSES is a promising tool (Payne & Jahoda, 2004). However, the GSSES is rarely utilized, and self-developed tools are often used. For instance, a modified physical self-efficacy scale, not the GSSES, was employed after implementing an exercise program (Jo et al., 2018). Another study examined the relationship between career interests and self-efficacy beliefs, which were measured by My Future Preferences (Nota et al., 2010). One study found that fitness and health education significantly increased exercise self-efficacy among adults with Down syndrome based on an original scale (Heller et al., 2004).

The General Self-Efficacy Scale (GSES) is the most commonly used instrument for measuring self-efficacy (Schwarzer et al., 1997). This widely used ten-item scale has shown good internal consistency, with values of 0.84 for German students, 0.81 for Costa Rican students, and 0.91 for Chinese student samples (Schwarzer et al., 1997). In South Korea, the GSES has been used to collect large-scale panel data. For instance, the Korea Employment Agency for Persons with Disabilities has complied an Employment Panel Survey of Persons with Disabilities, which serves as a representative dataset of persons with ID. However, the psychometric properties of the GSES for individuals with ID have not been reported.

Factor analysis is a classical method of validating the psychometric properties of instruments. This statistical technique extracts common factors based on the correlations among variables, and is useful for confirming the existence of latent factors. Previous studies have primarily used factor analysis to verify the validity of the GSES and confirm its factor structure (Nel & Boshoff, 2016; Schwarzer et al., 1997). However, factor analysis has several limitations. First, researcher subjectivity is likely to be involved in determining the number of factors and their interpretations, which can lead to inconsistent results (Costello & Osborne, 2005). Second, reliable results are difficult to obtain without a sufficient sample size, which typically requires at least five to ten times the number of variables (Fabrigar & Wegener, 2012). Third, researcher judgment is required in selecting appropriate variables, which can impact the analysis results (DeVellis & Thorpe, 2021). Fourth, factor analysis assumes linear relationships between variables, making it difficult to reflect complex non-linear relationships (Preacher & MacCallum, 2003). These limitations hinder the ability to ensure the reliability and validity of measurement tools using factor analysis alone.

In contrast, the item response theory (IRT) is a statistical technique that analyzes subject ability and item characteristics at the individual item level, thereby enabling more precise measurements. First, it allows for the assessment of item difficulty, discrimination, and predictability, ensuring objective analyses of individual item quality (Embretson & Reise, 2013). Second, unlike factor analysis, which relies on the overall score of a subject, the IRT independently measures items and subject ability, providing a reliable tool across diverse populations (Baker & Kim, 2004). Third, the IRT allows computer-adaptive testing (CAT), which provides items tailored to the respondent’s abilities, enabling highly reliable measurements within a short period of time (Embretson & Reise, 2013). Fourth, this method eliminates unnecessary items and shortens the questionnaire length while maintaining high measurement accuracy, thereby reducing respondent fatigue and maximizing efficiency (Van der Linden & Glas, 2000). Finally, the IRT is not limited to a specific group; the same scale can be applied to various groups, thereby increasing the generalizability of the findings (Embretson & Reise, 2013). Due to these advantages, the IRT is increasingly used in assessment tool development and scale validation.

Therefore, this study aimed to investigate the psychometric properties of the GSES among individuals with ID using Rasch analysis based on the IRT. This study posed the following research questions:

1. What is the item fit of the GSES?
2. What is the item difficulty of the GSES?
3. What is the fit of the GSES rating scale?
4. What are the person and item separation indices of the GSES?

## 2. Materials and Methods

### 2.1. Data

This study analyzed data from the first year of the second wave of the Employment Panel Survey of Persons with Disabilities provided by the Korea Employment Agency for Persons with Disabilities. The purpose of this panel was to produce dynamic basic statistics on the overall economic activities of people with disabilities and to identify the personal and environmental factors that influence these activities, thereby providing useful baseline data for establishing and evaluating employment policies for people with disabilities. The first survey was conducted in 2016, targeting persons with disabilities registered under the Disabled Persons Welfare Act (aged 15–64) residing in Korea as of 15 May 2016. Stratified sampling was used first to divide the population according to various characteristics, such as residential area, type of disability, disability severity, and age. Next, probability proportional sampling was used to recruit participants.

This study used the Second Wave of the Panel Survey on Employment of Persons with Disabilities. The legal basis of this panel survey is Article 26 of the Act on Promotion of Employment and Vocational Rehabilitation of Persons with Disabilities (Survey on the Status of Persons with Disabilities). The survey was approved under Article 18 of the Statistics Act (Approval for Compilation of Statistics), with approval number 383003. All confidential information to individuals, corporations, or organizations was used only for the purpose of compiling statistics and deleted during this process, in accordance with Chapter 33 of the Statistics Act. As per Chapter 16 of the Bioethics Law of Korea and Chapter 13 of the Enforcement Regulation, IRB approval is not required for research that uses public information or does not collect or record personal identification information.

A total of 398 individuals with ID were identified in the dataset. After excluding 166 individuals with incomplete responses; a total of 232 participants with ID were included in the final analysis. In Rasch analysis, the key criterion for determining the reliability of item calibration is the standard error (SE). When three criteria are met—sufficient sample size (*N*), respondents appropriately distributed across item levels, and items answered in the intended manner—the average correct response rate for an item generally falls within the range of 0.50 to 0.87. The stability of item difficulty is determined by the sample size, with the SE decreasing as the sample size increases. It has been suggested that the SE of an item in the Rasch model theoretically lies between 2/√*N* and 3/√*N* (B. D. Wright & Stone, 1979). Furthermore, to reliably estimate item difficulty, at least eight correct and eight incorrect responses—i.e., responses from participants with different characteristics—are required for each item. For item difficulty to be accurately estimated within ±1 logit, the SE must be approximately 0.38 logits or less, calculated based on a 99% confidence interval (±2.6 SE). Converting this to sample size, the minimum sample size for stable item difficulty estimation is between 27 and 61. A sample size as low as 30 is considered appropriate for preliminary or exploratory analyses (B. Wright & Panchapakesan, 1969). Therefore, this study’s sample size of 232 participants is sufficient to perform Rasch analysis and reliably estimate item difficulty.

The participant characteristics are presented in Table 1. The participants comprised more men than women, and most participants were aged 15–29 years. In terms of disability severity, 50% of participants were classified as grade 3, which corresponds to relatively mild severity. Furthermore, most participants had a high school diploma and had no comorbid disabilities. Regarding the types of comorbid disabilities, mental illness accounted for the highest proportion (6 people, 26.1%), followed by autism spectrum disorder (4 people, 17.4%); physical disability, brain damage, and speech impairment (3 people each, 13.0%); and visual impairment and epilepsy disorder (2 people each, 8.7%) each. Those without comorbid disabilities accounted for 90.1% of the total. The distribution of residences was relatively even across large cities, small or medium-sized cities, and rural areas. Moreover, 80.2% of the participants were unmarried.

### 2.2. Measure

Self-efficacy was measured using the GSES developed by Jerusalem and Schwarzer (2014). The scale comprised the following questions: (1) “I believe that if I try hard enough, I can achieve it”; (2) “I find it relatively easy to focus on my goals”; (3) “I am not embarrassed by difficulties because I believe in my abilities”; (4) “I can solve most problems if I put in the necessary effort”; (5) “I believe that I will find a way to solve difficulties when they arise”; (6) “I believe that I will eventually be able to do a task effectively, even if I did not expect the task at first”; (7) “I can deal well with unexpected situations using my abilities”; (8) “When problems arise, I usually find a solution”; (9) “Even if someone disagrees with my opinion, I can still find a way to do it the way I want”; and (10) “I believe that I can handle it, no matter what happens.” Scores were calculated as the mean of ten items. Responses were rated on a four-point Likert scale (1 = strongly disagree; 4 = strongly agree), with higher scores indicating higher self-efficacy. Multicultural validation studies with South Korean respondents have reported consistent evidence of associations between perceived self-efficacy and the variables under study, confirming the validity of the GSES (Luszczynska et al., 2005). The Cronbach’s α of the scale was 0.995 in this study. This high value indicates that the GSES items may be too similar.

### 2.3. Statistical Analysis

The GSES item fit was assessed using Rasch analysis. Rasch analysis is used to confirm the unidimensionality of a scale (Smith & Miao, 1994). Unidimensionality assumes that the concept of being measured is composed of a single attribute or factor. The scale can be considered to accurately measure the concept being analyzed, and the total score can be calculated using the scale, only if the assumption of its unidimensionality is confirmed. Various studies have examined whether the scales developed for the general population demonstrate unidimensionality in other clinical groups. This study employed the rating scale model of Rasch analysis (Andrich, 2016; Battisti et al., 2010). To evaluate the fit to the unidimensionality of Rasch analysis and whether each item explained the common construct, this study used two item fit indices: infit (weighted) and outfit (unweighted) mean-square (MNSQ) values. The ideal fit value is 1.0 (Bond & Fox, 2013). Linacre et al. (2002) suggested a maximum allowable value of 1.5. Since infit MNSQ is sensitive to response patterns and difficult to diagnose, which could be more greatly treat to measurements. Therefore, this study considered items unsuitable if the infit MNSQ value was less than 0.6 or greater than 1.4 (Bond & Fox, 2013).

The difficulty of the GSES items was assessed using the Wright map of Rasch analysis as an indicator of construct validity. This tool visually shows the distribution of respondents and items; respondents located at the top indicate people with high levels of the corresponding construct concept, whereas items located at the top indicate items that are most difficult for respondents. If the average of the respondents was higher than the average of the items, the item was interpreted as relatively easy among respondents; conversely, if it was lower, the item was interpreted as difficult (Bond & Fox, 2013).

To examines the appropriateness of the four-point Likert scale used for the GSES, a categorical functional analysis was conducted on the Rasch model. The scale was evaluated using the mean, structural value, infit and outfit MNSQ of each category, and thresholds. The mean and structural values were expected to increase as the score increased; if the fit of each category was 1.5 or higher, the function was considered inadequate (Andrich, 2016). If the threshold did not progress linearly, the construct concept was considered not well reflected. Winsteps 3.6 was used for the analysis.

The person and item separation indices were used to describe the reliability of the questionnaire for Rasch analysis. A higher separation index indicated that the questionnaire could identify more distinct functions. A separation index higher than 2.0 was considered to reflect a good level of separation. Separation reliability was interpreted the same as Cronbach’s α (Boone et al., 2014).

## 3. Results

### 3.1. Unidimensionality

The results of item appropriateness are presented in Table 2, Table 3 and Table 4. The item fit test determined the second item (“I find it relatively easy to focus on my goals”) to be unfit. After the second item was deleted, and the scale was reanalyzed, the third item (“I am not embarrassed by difficulties because I believe in my abilities”) was determined to be unfit. Finally, eight items were evaluated as having an appropriate fit.

### 3.2. Item Difficulty

**Figure 1:**
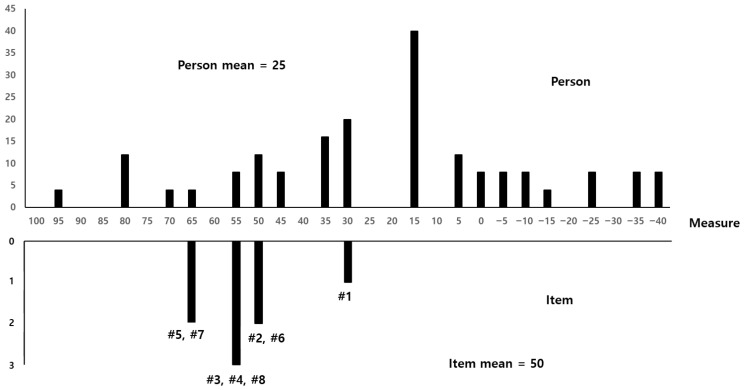
Item difficulty map. # means item number.

Figure 1 shows a comparison of item difficulty and individual abilities. The item mean was higher than the person means, indicating that some items were more difficult than the abilities of individuals with ID.

### 3.3. Suitability of the Rating Scale

The four-point Likert scale was suitable for measuring self-efficacy in individuals with ID (Table 5). The infit and outfit MNSQ values for each category were smaller than 1.5, and the structure measure increased with increasing levels.

### 3.4. Person and Item Separation Indices

The results of the person and item separation indices are presented in Table 6. Both separation indices were greater than 2.0, indicating good reliability.

## 4. Discussion

This preliminary study aimed to identify the need and direction for developing a self-efficacy measurement tool for people with ID by analyzing the reliability and validity of the GSES based on the Rasch model. The results demonstrated the unidimensionality of the GSES for individuals with ID. The results demonstrated the unidimensionality of the GSES for individuals with ID. These findings not only confirm the psychometric validity of the GSES for this population but also deepen understanding of how the cognitive and contextual characteristics of individuals with ID influence their perceptions of self-efficacy. The item fit analysis revealed that Items 2 and 3 did not conform to the expectations of the Rasch model. This suggested that the cognitive characteristics of individuals with ID and their limitations in abstract self-awareness affected their interpretation of items and response consistency (McGrew & Evans, 2004; Schalock et al., 2021). These findings were consistent with those of Park and Park (2019), who analyzed the Rosenberg Self-Esteem Scale for individuals with ID and demonstrated the utility of the Rasch model for measuring the psychological characteristics of individuals with developmental disabilities, revealing a low fit for items containing abstract concepts. Measuring self-efficacy for individuals with ID requires considering both cognitive demands, such as sentence structure and readability, and contextual relevance, such as daily life or familiar environments. Therefore, strategies such as specificity, short sentence structure, and experience-based statements are required when selecting item wording (Kim & Gray, 2024). Alternative expressions, particularly for items with high interpretability, should be developed.

Jo et al. (2018) demonstrated that researchers can secure the reliability of measurement tools by considering differences in the comprehension abilities of individuals with ID and implementing explanation and response verification procedures. These results suggest the need for alternative wording and support procedures to enhance the interpretability of items in surveys targeting individuals with ID. In particular, panel surveys that include individuals with ID should consider an approach similar to that of Jo et al. to enhance data reliability, thereby enhancing the validity and usability of the results. Furthermore, the results revealed that the difficulty of the GSES exceeded the average abilities of individuals with ID. This finding provides valuable insight into how currently standardized psychometric tools inadvertently underestimate the strengths of individuals with ID. This was likely because the original scale was developed based on the general population (Hampton & Mason, 2003). The confidence and self-efficacy that individuals with ID may possess in authentic or familiar settings may not be adequately captured by commonly used scales reliant on general statements that require metacognitive reflection. Therefore, the problem may not be low self-efficacy but rather a mismatch between the item format and actual experiences. Therefore, individuals with ID may have struggled with the levels of cognitive reasoning or emotional self-insight required by the GSES items. Similarly, Park and Park (2019) reported that discrepancies occurred between item difficulty and subject characteristics when existing scales did not sufficiently account for the developmental characteristics of individuals with ID. This finding is consistent with the developmental direction of the University of Washington Self-Efficacy Scale for individuals with disabilities, which uses items structured around the daily life and functions of individuals with disabilities, thereby enhancing the scale’s reliability and validity by presenting realistic items aligned with the respondents’ experiences (Amtmann et al., 2012). This highlights the importance of adopting a strengths-based assessment model (Thompson et al., 2016) that emphasizes the expression of self-efficacy based on experience and context.

Although the four-point Likert scale elicited relatively stable responses, suggesting its applicability for people with ID, the finding that only 1% of participants selected the highest option suggests that it may not have clearly understood or appropriately used. Finally, as the item and subject separation indices were greater than 4, the GSES possessed sufficient sensitivity to discriminate between individuals with ID. This demonstrated the stability of the Rasch-based scale, as defined by B. D. Wright and Masters (1982), and confirmed its ability to sensitively detect individual differences in self-efficacy among individuals with ID.

This study revealed that the GSES used in panel data could measure self-efficacy among individuals with ID. Most items showed good fit, confirming that they appropriately reflected the unidimensional structure of self-efficacy. However, certain adjustments are required. According to Bandura’s (1986) self-efficacy theory, individuals assess their abilities through a variety of cognitive appraisal processes, including task difficulty, prior success experiences, and environmental cues. The misfit of certain items in this study suggests that individuals with intellectual disabilities may engage in these cognitive processes differently from what the original GSES assumes, which helps explain the item-level misfit observed in the Rasch Model. Gobeille et al. (2024) conducted a Rasch analysis of the GSES for older adults with visual impairment and highlighted the need for cultural and contextual appropriateness of existing self-efficacy scales. Similarly, this study found that some items failed to reflect the complexity and contextual dependence inherent in self-efficacy, suggesting the need for designing items that fully reflect the life context of individuals with ID. The results of this study demonstrate that rather than simply applying existing theories to assess self-efficacy in people with intellectual disabilities, a theoretical restructuring that considers cognitive characteristics and information-processing methods is necessary. In other words, while maintaining the core elements of self-efficacy, the items assessing it need to be simplified, made more specific, and action-oriented. Self-efficacy is a fundamental psychological resource for individuals with ID in various domains, including independence, career development, and social participation. Therefore, it is essential to accurately measure their self-efficacy and use it as a basis for interventions. Building on previous studies that developed and applied My Future Preferences scale (Nota et al., 2010) and exercise self-efficacy scale (Heller et al., 2004), future research should develop a self-efficacy scale that distinguishes subdomains such as social and professional self-efficacy.

Although the use of panel data collected through a systematic sampling process represents a methodological strength, several limitations of this study should be noted. First, it used a rating scale originally developed for the general population. Because the items were not designed specifically for individuals with ID, some contained abstract wording and required high levels of cognitive and verbal reasoning. Therefore, some items may have been difficult for some respondents to understand accurately. As this was part of a panel survey that collected data from all individuals with disabilities, no alternative was available. Future research should develop tools to validate and reliably measure self-efficacy among individuals with ID. After developing a customized tool for people with ID, we hope to develop a method for utilizing the GSES in panel surveys that targets individuals with ID. Second, the generalizability of the sample was limited. While the sample size of 232 was sufficient for Rasch analysis, exceeding the recommended minimum of 30 to 100 participants (Linacre, 1994), a larger sample size reduces the SE and leads to more stable estimation. To address this, further large sample size studies are needed to generate more stable and reliable results. Moreover, this study used data from a panel study targeting individuals aged 15 years and older, which prevents generalizing the results to the entire population of individuals with ID. The GSES should be tested in practical contexts using a representative sample of individuals with ID of various ages to develop a more effective tool for this population. Third, bias may have been present in the self-reported responses. Of the 398 individuals with ID, only 232 were able to self-report and were included in the analysis. The remaining 166 were excluded from the analysis due to missing data. This may lead to an over-representation of the characteristics of individuals with milder ID who could self-report. Future studies should confirm the validity of the tool for people with severe ID, using additional support materials. Lastly, because this study was conducted in Republic of Korea, the results reflect cultural characteristics unique to the South Korean culture, such as the disability support system Therefore, caution is needed when interpreting the findings and considering their applicability to other countries and regions. In this regard, cross-cultural studies are recommended.

## 5. Conclusions

This study analyzed items measuring self-efficacy in individuals with ID using the Rasch model. After deleting two of the ten items, the remaining eight items of the GSES showed good fit, confirming that they adequately reflected the unidimensional structure of self-efficacy. The results of this study suggest that when analyzing the self-efficacy of people with ID using national panel data, it is more appropriate to use only the eight items verified through Rasch analysis, rather than the results from all ten items. Self-efficacy is a key psychological resource for individuals with ID in various aspects, including independence, career development, and social participation. Therefore, precisely measuring self-efficacy and utilizing it as a basis for interventions is crucial. Future research should develop a self-efficacy scale that distinguishes subdomains, such as social and career self-efficacy. Furthermore, this scale should be developed using a representative sample of individuals with ID of various ages and validated in practical settings to develop a more effective tool.

## Figures and Tables

**Table 1:** Participant characteristics.

Category	n	%	*X* ^2^
Gender			
Men	147	63.4	18.589 **
Women	85	36.6	
Age (years)			
15–29	107	46.1	136.879 **
30–39	63	27.2	
40–49	29	12.5	
50–59	29	12.5	
60–64	4	1.7	
Education level			
No education	16	6.9	352.017 **
Elementary school graduate	26	11.2	
Junior high school graduate	27	11.6	
High school graduate	144	62.1	
College graduate	11	4.7	
University graduate	8	3.4	
Severity of disability			
Grade 1	40	17.2	27.379 **
Grade 2	78	32.8	
Grade 3	116	50.0	
Comorbid disability			
Yes	23	9.9	149.121 **
No	209	90.1	
Residential area			
Big city	83	35.8	6.060 *
Small or medium-sized city	60	25.9	
Rural area	89	38.4	
Marital status			
Unmarried	186	80.2	383.017 **
Married	31	13.4	
Divorced	10	4.3	
Bereaved	5	2.2	

Note. * *p* < 0.05; ** *p* < 0.001; n = number of participants; Grade 1 = IQ < 35 and requires lifelong supervision; Grade 2 = IQ 35–49 and requires continuous supervision or specific training; Grade 3 = IQ 50–70 and capable of social and vocational participation with appropriate support.

**Table 2:** Item fit statistics for ten items.

Item No.	Measure	SE	Infit		Outfit	
			MNSQ	Z-Value	MNSQ	Z-Value
1	31.21	1.70	1.23	2.1	1.39	2.8
2 *	51.89	1.75	1.81	6.0	1.69	4.2
3	49.15	1.74	1.24	2.1	1.16	1.2
4	51.89	1.75	0.83	−1.6	0.72	−2.2
5	52.19	1.75	0.85	−1.4	0.75	−1.9
6	51.58	1.75	0.96	−0.4	0.91	−0.6
7	54.13	1.76	0.87	−1.2	0.87	−0.9
8	50.67	1.75	0.65	−3.7	0.56	−3.8
9	54.66	1.76	0.69	−3.1	0.60	−3.3
10	52.62	1.76	0.73	−2.7	0.64	−3.0

Note*:* * Items did not fit the Rasch model (infit MNSQ outside the 0.60–1.40 range). SE, standard error; MNSQ, mean square.

**Table 3:** Item fit statistics for nine items.

Item No.	Measure	SE	Infit		Outfit	
			MNSQ	Z-Value	MNSQ	Z-Value
1	30.95	1.61	1.24	2.4	1.34	2.5
3 *	48.92	1.66	1.59	5.0	1.45	3.0
4	51.41	1.67	0.96	−0.4	0.87	−0.9
5	52.25	1.67	0.82	−1.8	0.69	−2.5
6	52.53	1.67	1.00	0.0	0.94	−0.4
7	54.60	1.69	1.02	0.3	1.05	0.4
8	51.97	1.67	0.67	−3.6	0.55	−3.9
9	54.22	1.68	0.75	−2.6	0.65	−2.8
10	53.17	1.68	0.74	−2.7	0.64	−2.9

Note*:* * Items did not fit the Rasch model (infit MNSQ outside the 0.60–1.40 range). SE, standard error; MNSQ, mean square.

**Table 4:** Item fit statistics for eight items.

Item No.	Measure	SE	Infit		Outfit	
			MNSQ	Z-Value	MNSQ	Z-Value
1	29.46	1.69	1.33	3.0	1.50	3.2
4	51.55	1.72	1.27	2.5	1.16	1.1
5	51.85	1.72	0.93	−0.6	0.85	−1.0
6	51.85	1.72	1.01	0.1	0.93	−0.5
7	55.19	1.74	0.92	−0.8	0.86	−0.9
8	51.55	1.72	0.82	−1.9	0.72	−2.1
9	56.02	1.74	0.70	−3.2	0.59	−3.2
10	52.53	1.73	0.78	−2.3	0.68	−2.4

Note: SE, standard error; MNSQ, mean square.

**Table 5:** Rating scale analysis of the GSES.

Category Level	Observed Count	Observed Rate (%)	Average Measure	Infit MNSQ	Outfit MNSQ	Structure Measure
1	225	16	−75.18	0.84	0.67	None
2	807	57	−31.98	0.93	0.98	−67.33
3	365	26	12.06	1.07	1.07	−2.75
4	17	1	55.95	1.31	1.40	70.07

**Table 6:** Person and item separation indices of the GSES.

Category	Separation Index	Reliability
Person	4.33	0.95
Item	4.50	0.95

## Data Availability

Data can be requested at: https://edi.kead.or.kr/BoardType17.do?bid=18&mid=37 (accessed on 25 November 2024).

## Abbreviations

GSES	General Self-Efficacy Scale
ID	Intellectual disabilities
GSSES	Glasgow Social Self-Efficacy Scale
IRT	Item response theory
CAT	Computer-adaptive testing
MNSQ	mean square
